# Case Report: Iatrogenic left main coronary artery dissection during premature ventricular contraction ablation managed with emergent coronary artery bypass grafting

**DOI:** 10.3389/fcvm.2026.1943586

**Published:** 2026-09-15

**Authors:** Michelle C. Carvajal, Shivani Shirodkar, Emmanuel Robinson, Jeff Trost, Sunil K. Sinha, Jennifer S. Lawton

**Affiliations:** 1 Division of Cardiac Surgery, Department of Surgery, Johns Hopkins University School of Medicine, Baltimore, MD, United States; 2 Division of Cardiology, Department of Medicine, Johns Hopkins University School of Medicine, Baltimore, MD, United States

**Keywords:** case report, catheter ablation, coronary artery bypass grafting (CABG), intravascular ultrasound (IVUS), left main coronary artery dissection, premature ventricular contraction (PVC)

## Abstract

Premature ventricular contraction (PVC) ablation via retrograde aortic access is generally safe, but iatrogenic left main coronary artery (LMCA) dissection is a rare and potentially catastrophic complication. This case adds to the literature by describing LMCA dissection during PVC ablation managed with primary emergent coronary artery bypass grafting (CABG) rather than percutaneous coronary intervention (PCI), guided by intravascular ultrasound (IVUS) and Heart Team decision-making. A 60-year-old man with symptomatic, medication-refractory monomorphic PVCs (17.4% burden with nonsustained ventricular tachycardia) and preserved left ventricular function underwent elective PVC ablation via retrograde aortic access. Shortly after successful ablation at the aortomitral continuity, he developed sudden excruciating chest pain and anterior ST-segment elevation with hemodynamic instability. Emergent coronary angiography suggested LMCA dissection with occlusion of the proximal left anterior descending (LAD) artery and near-occlusive involvement of the proximal ramus intermedius. Balloon angioplasty restored limited flow, and intravascular ultrasound (IVUS) demonstrated an ostial LMCA dissection extending into both branches with true lumen compression and a heavily calcified proximal LAD segment, making complex LMCA bifurcation stenting high risk. After rapid Heart Team consultation, the patient underwent emergent CABG with left internal mammary artery (LIMA)–LAD and saphenous vein grafts to the ramus intermedius branch of the LMCA, along with intra-aortic balloon pump support. He was extubated and weaned off mechanical support on postoperative day 1 and discharged home on day 8; follow-up at 3 months revealed reduced ejection fraction (25%–30%) managed by advanced heart failure services. This case highlights mechanical LMCA ostial injury as a rare complication of retrograde PVC ablation and highlights the importance of immediate recognition of ST-segment elevation, emergent coronary angiography, and early involvement of a multidisciplinary Heart Team. IVUS is crucial to define dissection extent. When ostial or bifurcation LMCA dissection occurs in the setting of hemodynamic compromise and calcified, stent-unfavorable anatomy, guideline-directed emergent CABG can provide definitive and potentially life-saving revascularization.

## Introduction

Premature ventricular contraction (PVC) ablation is an established and generally safe therapy for symptomatic ventricular ectopy refractory to medical management, with major complication rates below 1% (1). Coronary artery injury is a recognized but rare complication of catheter ablation, occurring most frequently in the context of atrial fibrillation procedures due to thermal injury or catheter trauma in proximity to coronary ostia (2). Iatrogenic left main coronary artery (LMCA) dissection specifically during PVC ablation is rare. A systematic review identified only 22 cases of LMCA injury during any catheter ablation through 2018 (2), nearly all during atrial fibrillation procedures.

We report a case of acute anterior ST-elevation myocardial infarction (STEMI) caused by iatrogenic LMCA dissection extending into the proximal LAD artery and the proximal ramus intermedius branch during PVC ablation via retrograde aortic access, managed successfully with emergent CABG. This case emphasizes the mechanism of coronary ostial injury during retrograde aortic approach, the critical role of intravascular ultrasound (IVUS) in characterizing dissection anatomy and guiding revascularization strategy, and the importance of rapid multidisciplinary Heart Team activation.

Although left main coronary artery dissection during PVC ablation has been previously reported, published cases have predominantly been managed with percutaneous stenting (2). To our knowledge, we found no previously published English-language report of iatrogenic LMCA dissection during catheter ablation managed with primary emergent CABG as the definitive revascularization strategy, without antecedent left main stent placement or failed left main PCI.

## Case description

### Patient presentation and preprocedural evaluation

A 60-year-old male presented for elective PVC ablation for symptomatic, medication-refractory monomorphic ventricular ectopy. Holter monitoring demonstrated a 17.4% PVC burden with 46 episodes of nonsustained ventricular tachycardia (longest run 5 beats). His medical history was significant for paroxysmal atrial fibrillation and atrial flutter, status post two prior pulmonary vein isolation procedures, hypertension, dyslipidemia, obesity, obstructive sleep apnea, type 2 diabetes mellitus, and prostate cancer treated with surgery and radiation. His CHA_2_DS_2_-VASc score was 3. Preprocedural coronary CT angiography demonstrated marked calcification of the proximal LAD artery (Figure 1). Transthoracic echocardiography confirmed preserved left ventricular ejection fraction (55%) with normal wall thickness. Cardiac catheterization demonstrated a 60% stenosis of the distal right coronary artery (RCA).

**Figure 1:**
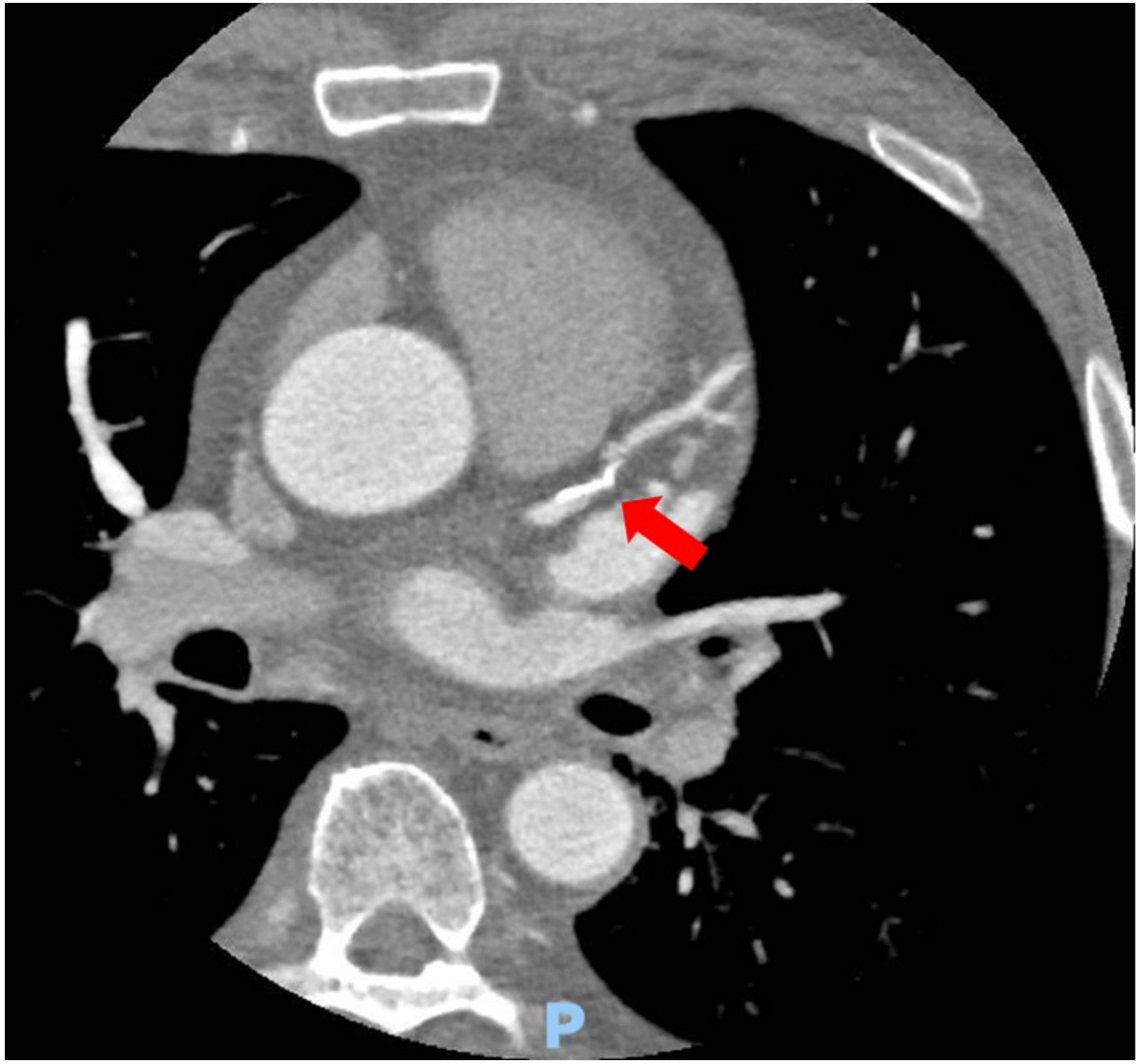
Preprocedural coronary computed tomography angiography. Axial contrast-enhanced coronary CT angiogram obtained prior to elective catheter ablation shows marked calcification of the proximal left anterior descending (LAD) artery (red arrow), indicating pre-existing atherosclerotic disease of the LAD.

### Ablation procedure and acute complication

The procedure was performed with the patient under minimal sedation to preserve PVC inducibility. Right femoral venous and arterial sheaths were placed under ultrasound guidance, and therapeutic anticoagulation was achieved with intravenous heparin.

Electroanatomic mapping of the clinical PVC using the Pentaray mapping catheter (CARTO system) via retrograde aortic access localized the earliest activation to the aortomitral continuity of the left ventricle. The mapping catheter was exchanged for a QDOT MICRO ablation catheter (Biosense Webster), and radiofrequency energy at 50 watts was delivered to the aortomitral continuity target site. Clinical PVCs persisted, prompting re-mapping with the Pentaray catheter; a second site, adjacent to the initial aortomitral continuity target, was identified and ablated at 50 watts. No further clinical PVCs were observed after the second set of radiofrequency applications.

Within the first minute following the final radiofrequency energy delivery, the patient developed sudden excruciating chest pain with anterior ST elevation and inferior reciprocal changes on continuous monitoring. The isoproterenol infusion was immediately stopped, and the interventional cardiology team was emergently notified and at bedside within minutes.

### Diagnostic evaluation

Emergent coronary angiography demonstrated occlusion of the proximal LAD and 99% stenosis of the proximal ramus intermedius branch, with subtle haziness at the LMCA suggestive of dissection (Figure 2A). The RCA demonstrated a 60% distal stenosis.

**Figure 2:**
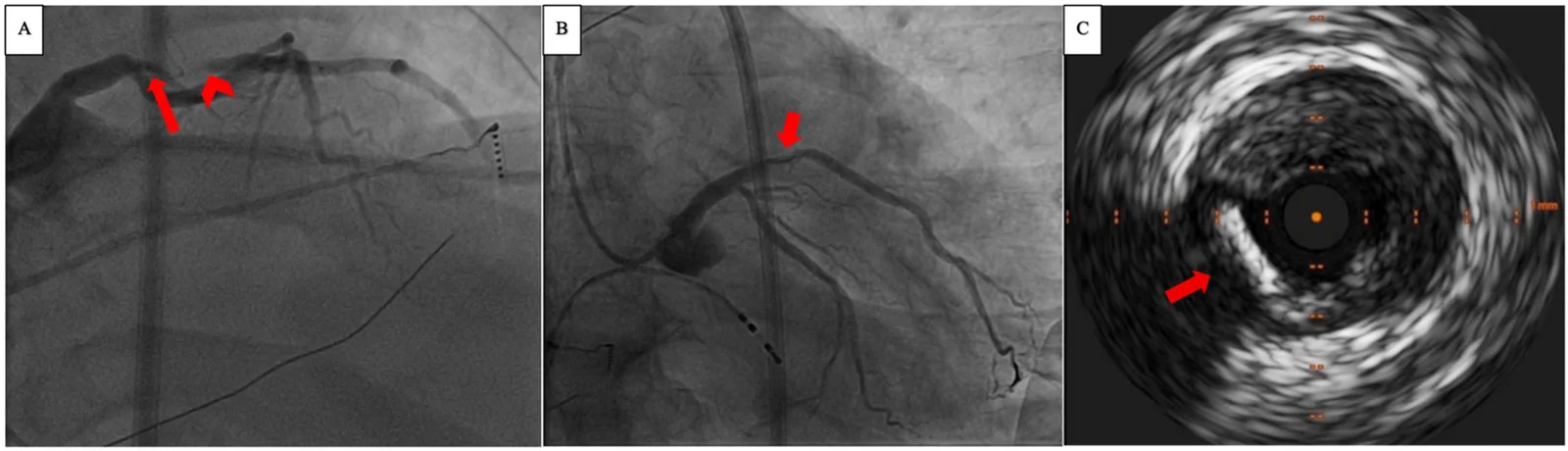
Coronary angiography and intravascular ultrasound (IVUS). **(A)** Right anterior oblique caudal view showing subtle haziness/luminal narrowing at left main coronary artery (LMCA) (arrowhead), occlusion of the proximal LAD (long arrow). **(B)** Post-balloon angioplasty angiogram showing persistence of the LMCA narrowing and ongoing LAD non-filling despite balloon inflation. **(C)** IVUS cross-sectional frame demonstrating the dissection flap (arrow) within the LMCA with true lumen compression.

Balloon angioplasty of the LAD was performed, followed by IVUS using an Eagle Eye Platinum catheter (Philips) with pullback from the LAD through the LMCA (see Supplementary Video S1). IVUS confirmed a dissection flap within the LMCA extending into the proximal LAD and compromising flow to the proximal ramus intermedius branch (Figures 2B,C). The patient was intubated and transesophageal echocardiography excluded involvement of the aortic arch, ruling out type A aortic dissection. An 8-French, 50-cc intra-aortic balloon pump (IABP) was inserted in the right femoral artery with improvement in blood pressure.

### Therapeutic intervention

Real-time Heart Team consultation among the electrophysiology, interventional cardiology, and cardiac surgery teams was convened. Multiple percutaneous strategies were considered, including provisional stenting, two-stent bifurcation techniques, cutting-balloon fenestration, and covered stent placement, and each was rejected based on the IVUS-defined anatomy. Ostial dissection extending across the bifurcation into both the LAD and ramus origins with hemodynamic compromise and proximal LAD calcification made the lesion unfavorable for stent expansion.

Emergent CABG offered complete surgical exclusion of the dissected LMCA segment, durable revascularization of all jeopardized territories, and avoidance of stent placement within a hematoma-filled vessel. The patient was transferred to the operating room approximately 104 minutes after symptom onset, inclusive of IVUS, transesophageal echocardiography, and IABP placement.

### Operative management

The patient arrived to the operating room intubated and invasive monitoring lines already in place, allowing emergent surgical preparation without delay. Median sternotomy was performed simultaneously with endoscopic saphenous vein harvest from the left lower extremity. Systemic heparinization was administered and cardiopulmonary bypass (CPB) was established via aortic and right atrial cannulation. CPB was initiated immediately given ongoing ST elevation, hypotension, and acute coronary ischemia. The left internal mammary artery (LIMA) was harvested in a skeletonized fashion. Catheters were placed in the ascending aorta and right atrium for antegrade and retrograde cardioplegia delivery, respectively. The aortic cross-clamp was applied and myocardial protection was achieved with 1 L of blood cardioplegia using warm induction, given the ongoing ischemia at the time of cross-clamping, followed by cold blood cardioplegia delivered via both antegrade and retrograde approaches.

Distal anastomoses were constructed sequentially. A saphenous vein graft (SVG) was anastomosed end-to-side to the posterior descending artery. A second SVG was anastomosed to the intramyocardial ramus intermedius branch. The LIMA was anastomosed to the LAD distal to the dissected segment. The left atrial appendage was amputated and oversewn in two layers of running Prolene suture. Two proximal anastomoses were constructed on the ascending aorta. The patient was rewarmed, de-airing maneuvers were performed, and the aortic cross-clamp was removed. The patient was weaned from cardiopulmonary bypass on epinephrine infusion with the IABP resumed at 1:1 augmentation. Total CPB time was 234 min; total aortic cross-clamp time was 101 min. Flow measurements confirmed acceptable patency in all conduits: SVG-posterior descending artery 176 mL/min (pulsatility index 3.7), SVG-ramus 30 mL/min (pulsatility index 1.8), and LIMA-LAD 16 mL/min (pulsatility index 4.2).

Left atrial appendage ligation was performed concurrently, supported by the patient's CHA_2_DS_2_-VASc score of 3 and established history of paroxysmal atrial fibrillation, consistent with guideline recommendations for left atrial appendage management during concomitant cardiac surgery in patients with atrial fibrillation at elevated thromboembolic risk (3). Key procedural and clinical metrics are summarized in Table 1.

**Table 1:** Procedural and clinical metrics.

Parameter	Value
PVC origin (electroanatomic mapping)	Aortomitral continuity, left ventricle
Ablation catheter	QDOT MICRO (Biosense Webster)
Radiofrequency energy delivered	50 watts (two separate aortomitral continuity sites)
Symptom onset (ST elevation)	Within 1 min of last radiofrequency delivery
IABP placed	8-Fr, 50cc
Patient transferred to operating room	∼104 min after symptom onset
Fluoroscopy time (AP/lateral)	21.4 min/10.3 min
IVUS catheter	Eagle Eye Platinum (Philips)
IVUS findings	Dissection flap LMCA extending into LAD and ramus intermedius; true lumen compression; entry at LMCA ostium
CPB time	234 min
Aortic cross-clamp time	101 min
Grafts	LIMA (skeletonized) → LAD: 16 mL/min, pulsatility index 4.2; SVG → posterior descending artery: 176 mL/min, pulsatility index 3.7; SVG → ramus intermedius (intramyocardial): 30 mL/min, pulsatility index 1.8
Peak troponin	36,820 ng/mL
Predischarge ejection fraction	40%–45% (anterior wall hypokinesis)
Hospital length of stay	8 days
Discharge rhythm	Sinus rhythm
CHA₂DS₂-VASc score	3 (hypertension, diabetes, vascular disease)

CABG, coronary artery bypass grafting; CPB, cardiopulmonary bypass; IABP, intra-aortic balloon pump; IVUS, intravascular ultrasound; LAD, left anterior descending artery; LIMA, left internal mammary artery; LMCA, left main coronary artery; PCI, percutaneous coronary intervention; PVC, premature ventricular contraction; RCA, right coronary artery; STEMI, ST-elevation myocardial infarction; SVG, saphenous vein graft.

### Postoperative course and follow-up

The patient was extubated and the IABP was removed on postoperative day 1. Peak troponin I reached 36,820 ng/mL, reflecting the substantial myocardial injury sustained during the approximately 6 h interval from symptom onset to restoration of coronary perfusion (aortic cross-clamp removal). His postoperative course was notable for recurrent paroxysmal atrial fibrillation treated with amiodarone and therapeutic anticoagulation. He was discharged home on postoperative day 8 in sinus rhythm.

Predischarge transthoracic echocardiography demonstrated a left ventricular ejection fraction of 40%–45% with anterior wall hypokinesis, consistent with the myocardial territory affected by the antecedent STEMI. At 3-month follow-up, transthoracic echocardiography demonstrated a decline in left ventricular ejection fraction to 25%–30%, and the patient was referred to advanced heart failure services for ongoing management. The clinical timeline is summarized in Figure 3.

**Figure 3:**
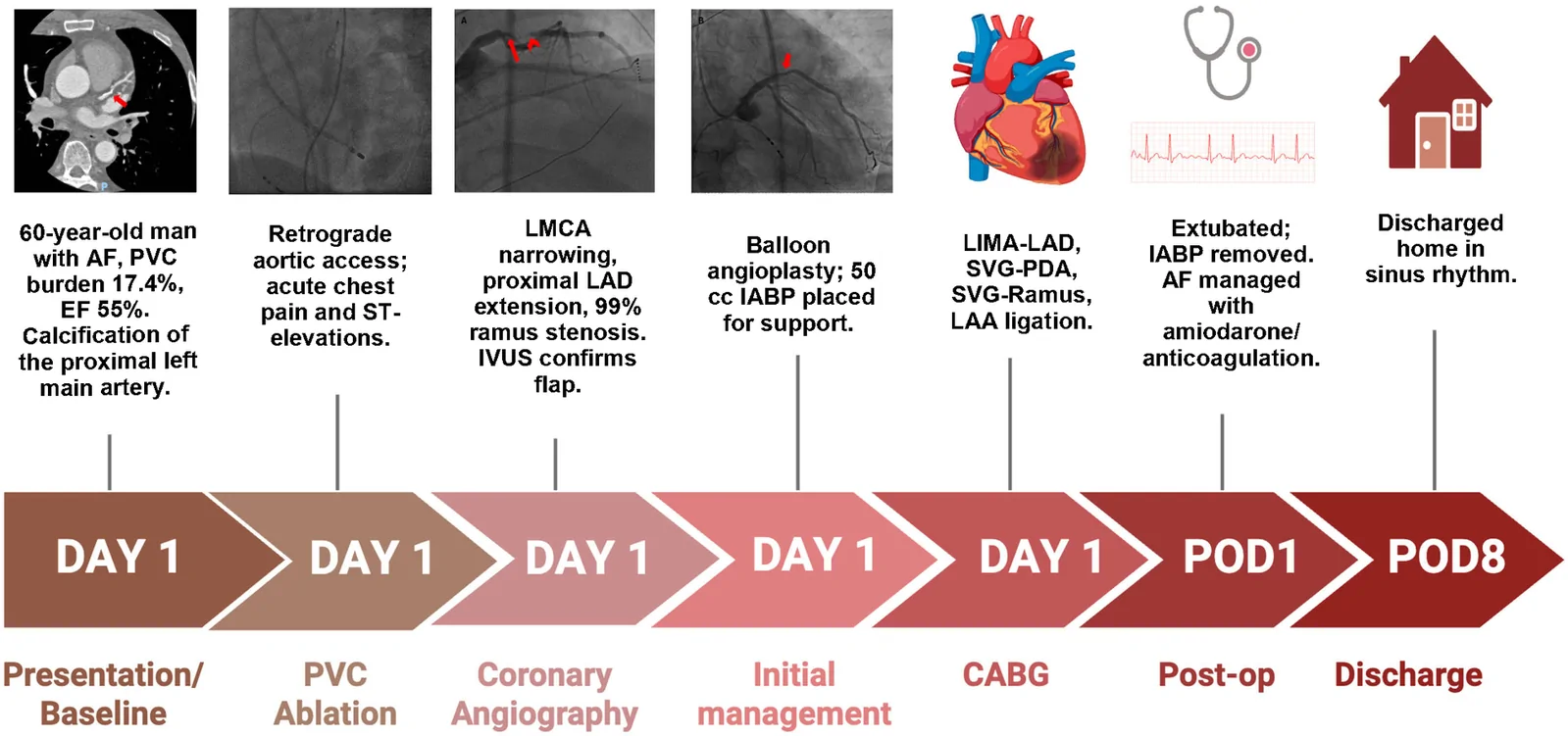
Clinical timeline. AF, atrial fibrillation; CABG, coronary artery bypass grafting; IABP, intra-aortic balloon pump; IVUS, intravascular ultrasound; LAA, left atrial appendage; LAD, left anterior descending artery; LIMA, left internal mammary artery; LMCA, left main coronary artery; PDA, posterior descending artery; POD, postoperative day; PVC, premature ventricular contraction; SVG, saphenous vein graft.

## Discussion

This case illustrates several teachable principles in the rare but devastating complication of iatrogenic LMCA dissection during PVC ablation. These include the specific mechanical mechanism of injury during retrograde aortic access, the indispensable role of IVUS in defining dissection anatomy, the structured rationale for surgical over percutaneous revascularization, and the life-saving value of immediate Heart Team coordination.

### Mechanistic considerations

In the retrograde aortic approach, the ablation catheter is advanced through the femoral artery across the aortic valve into the left ventricle for mapping and ablation of perivalvular ectopy. During catheter manipulation within the aortic root, particularly during withdrawal and torque maneuvers used to map the aortomitral continuity region, the catheter tip passes in direct proximity to the LMCA ostium, which originates approximately 10–20 mm above the aortic annulus (2, 7). Contact force applied during mapping or the recoil of the catheter upon valve crossing may generate sufficient mechanical stress to traumatize the ostial endothelium and initiate an intimal tear (4, 8). In this case, the mechanical nature of the injury is suggested by the absence of ablation delivery at or near the coronary ostium and the temporal relationship between catheter manipulation and symptom onset, rather than active radiofrequency application, a pattern distinct from the thermal injury mechanism described in atrial fibrillation ablation. IVUS findings, including the absence of significant plaque at the dissection site, support a traumatic rather than plaque-mediated mechanism, although the precise mechanical trigger cannot be definitively distinguished from the available imaging (4, 7, 8). Radiofrequency energy was delivered at the aortomitral continuity, remote from the LMCA ostium, whereas IVUS localized the dissection entry point to the ostium itself. The temporal association between symptom onset and the final RF application therefore reflects the timing of catheter manipulation within the aortic root rather than thermal energy transfer to the vessel. Although a thermal contribution cannot be entirely excluded, the evidence favors mechanical injury.

### The role of IVUS

Coronary angiography alone underestimates the severity and extent of coronary dissection, particularly at the LMCA where vessel geometry and overlapping structures can obscure a dissection flap. In the case presented, IVUS revealed that the dissection extended into both the proximal LAD and the ramus intermedius, a finding with direct therapeutic implications. The demonstration of true/false lumen anatomy and the bifurcation extent of the dissection established that no percutaneous strategy could safely address all of the involved myocardial territories. Clinicians encountering coronary dissection in the catheterization laboratory should consider the use of IVUS or optical coherence tomography when the extent or severity of dissection is uncertain. This diagnostic step enables rational, data-driven revascularization decision-making.

### Surgical versus percutaneous revascularization

Although no guidelines specifically address iatrogenic LMCA dissection, the general principles of STEMI management offer a reasonable framework. The 2021 ACC/AHA/SCAI Guideline for Coronary Artery Revascularization supports emergent CABG for STEMI with anatomy not amenable to percutaneous coronary intervention (PCI) (5), and the 2025 ACC/AHA/ACEP/NAEMSP/SCAI Guideline for the Management of Patients with Acute Coronary Syndromes reinforces individualized Heart Team decision-making for post-STEMI CABG when procedural complications preclude safe percutaneous management (6). Applying these principles to the dissection-specific literature, our case represents an uncommon scenario favoring CABG. In the presented patient's anatomy, an ostial LMCA dissection extending to both the LAD and ramus intermedius origins, hemodynamic compromise, and calcified proximal LAD unfavorable for stent expansion, exemplifies the guideline indication for surgical revascularization. CABG additionally enabled complete revascularization of the RCA territory given the known distal RCA stenosis and hemodynamic instability, a degree of completeness not achievable percutaneously in this acute setting. Published cases of LMCA dissection have emphasized percutaneous stenting when feasible (2, 4). In contrast, our case illustrates a scenario that favored emergent CABG as the primary revascularization strategy without LM stenting, with survival to discharge. This decision reflects individualized Heart Team judgment that would not apply to less extensive, stable dissections better suited to percutaneous stenting.

The concurrent 60% distal RCA lesion was grafted at the time of emergent surgery to achieve complete revascularization in a hemodynamically unstable patient already on cardiopulmonary bypass. Although routine bypass of intermediate lesions, particularly in the right coronary system, is of uncertain long-term benefit and may be subject to competitive flow, the low incremental risk of an additional distal anastomosis was judged acceptable in the setting of acute infarction, evolving left ventricular dysfunction, and mechanical support.

Limitations include the single-case design. Future prospective registry efforts to capture coronary complications of catheter ablation with systematic imaging documentation would substantially advance the evidence base for this rare complication.

## Conclusion

Iatrogenic LMCA dissection is a rare but potentially fatal complication that may occur during PVC ablation via retrograde aortic access through a direct mechanical mechanism at the coronary ostium. Sudden ST elevation during any ablation procedure demands immediate cessation of the procedure, emergent coronary angiography, and simultaneous activation of the cardiac surgery team. IVUS is essential to characterize the true extent of dissection and to define the best revascularization strategy. When left main bifurcation or trifurcation involvement, hemodynamic compromise, or anatomy unfavorable for stenting is identified, guideline-directed emergent CABG provides definitive and durable revascularization. This case underscores the importance of institutional readiness for immediate coronary angiography, intravascular imaging capability, and surgical backup for all retrograde access procedures.

### Take home points

- Retrograde aortic access for PVC ablation carries an underappreciated risk of mechanical LMCA ostial injury, likely related to catheter contact force during catheter manipulation in the aorta, distinct from the thermal injury mechanism of atrial fibrillation ablation. Sudden intraoperative ST elevation requires immediate coronary angiography and heart team coordination.
- IVUS is indispensable when coronary dissection is identified. Characterization of true/false lumen, dissection extent, and bifurcation/trifurcation involvement, are key to determine best revascularization strategy.
- Ostial, bifurcation, or trifurcation LMCA dissection with hemodynamic compromise represents a guideline-supported indication for emergent CABG.

## Patient perspective

The patient expressed anxiety regarding his own mortality after a cardiac CT demonstrated coronary artery disease prior to the elective catheter ablation for symptomatic premature ventricular contractions. In the postoperative period, he experienced clinical improvement with progressive gains in exertional capacity and is able to walk approximately half a block before stopping because of fatigue and dyspnea.

## Data Availability

The raw data supporting the conclusions of this article will be made available by the authors, without undue reservation.

## Supplementary material

Supplementary Video S1 Intravascular ultrasound (IVUS) pullback from the proximal LAD through the LMCA to the ostium using the Eagle Eye Platinum catheter.
